# Unlocking the blueprint to eliminating neglected tropical diseases: A review of efforts in 50 countries that have eliminated at least 1 NTD

**DOI:** 10.1371/journal.pntd.0013424

**Published:** 2025-09-04

**Authors:** Helmi Hietanen, Lorraine Tsitsi Pfavayi, Francisca Mutapi

**Affiliations:** 1 School of Biological Sciences, University of Edinburgh, King’s Buildings, Edinburgh, United Kingdom; 2 Institute of Immunology & Infection Research, University of Edinburgh, Ashworth Laboratories, Edinburgh, United Kingdom; 3 Tackling Infections to Benefit Africa (TIBA) Partnership, University of Edinburgh, Ashworth Laboratories, Edinburgh, United Kingdom; Consejo Nacional de Investigaciones Cientificas y Tecnicas, Fundación Mundo Sano, ARGENTINA

## Abstract

**Background:**

Neglected tropical diseases (NTDs) are a group of 21 diseases affecting approximately 1.5 billion people globally. Significant progress has been made in their control: by March 2024, 50 countries had eliminated at least one NTD, with 13 of these countries eliminating at least two. Togo achieved the highest milestone, having eliminated four. The eight NTDs eliminated in at least one country are Guinea worm disease, human African trypanosomiasis, lymphatic filariasis, onchocerciasis, rabies, trachoma, visceral leishmaniasis and yaws. We reviewed elimination efforts of these 50 countries to identify factors underlying their successes and failures to generate a blueprint to inform the acceleration of NTD elimination.

**Methodology/principal findings:**

We conducted a review of published and grey literature and extracted and recorded data on various features of the elimination programmes, including the durations and organisers of elimination efforts, interventions, strategies including mainstreaming into other health services, partnerships involved, and details of historical failed control efforts. These data were synthesised to generate a blueprint for NTD elimination. Key features of successful NTD elimination included country ownership, dedicated elimination efforts, and use of a combination of strategies. Most elimination programmes targeted one NTD at a time, while fewer utilised integrated approaches. Elimination required at least two decades of sustained efforts and partnerships between the endemic country and international stakeholders. Failure in historical efforts was frequently a result of sociopolitical instability, insufficient resources, deprioritisation, lack of effective interventions, or lax implementation of interventions.

**Conclusions/significance:**

Accelerating NTD elimination requires sustained, intense, and multisectoral approaches. In addition, mainstreaming within the health system, improved cross-cutting One Health strategies including water, sanitation and hygiene, and sustained financing are critical for elimination. While this study provides valuable insights, limitations due to documentation gaps and secondary sources highlight the need for improved data reporting and future research to strengthen elimination frameworks.

## Author summary

The World Health Organization has set a target of 100 countries each eliminating at least one neglected tropical disease (NTD) by 2030. By March 2024, we had reached half that target with 50 countries each eliminating at least one NTD. These countries provide an opportunity to learn lessons that can inform elimination efforts in other countries. Thus, we have studied the NTD elimination programmes of these 50 countries to develop a blueprint detailing common characteristics of successful and unsuccessful NTD elimination efforts. We identified the following as key components of successful elimination: country ownership of the programmes, dedicated elimination programmes, and development partner support. Our investigation also showed that elimination required sustained efforts, with elimination frequently taking approximately two decades. Most elimination programmes targeted a single NTD at a time. Moreover, the study showed the need for intensification of cross-sectoral interventions including water, sanitation and hygiene (WASH), prioritisation of NTD control in national health strategies, and increased domestic and international funding. Failed historical elimination efforts were characterised by disruption due to various factors including socio-political instability and insufficient resources. This underscores the importance of sustained and well-financed elimination programmes.

## Introduction

Neglected tropical diseases (NTDs) are a group of 21 preventable and treatable diseases [1,2] caused predominantly by a variety of pathogens including viruses, bacteria, fungi, helminths, protozoan and ecto-parasites, and toxins [3–5]. NTDs afflict an estimated 1.5 billion people globally [2,6], and result in the annual loss of approximately 19 million disability-adjusted life years (DALYs) [7]. Consequences of NTDs include disfigurement, debilitation and disability, which often lead to stigmatisation, discrimination, productivity losses, and hindered educational and socioeconomic advancement [4,7].

More widely, NTDs impede progress towards several of the targets of the United Nations’ (UN) poverty, health and education Sustainable Development Goals (SDGs) [4,8,9]. For example, annual NTD-related losses of household wages and income are estimated at 33 billion US dollars [10]. According to the World Health Organization (WHO), eliminating NTD-related household health expenses and lost productivity would result in a benefit of over 342 billion US dollars between 2015 and 2030. Efforts to combat NTDs are widely regarded as ‘best buys’ in both global health and development as investment in NTD control and elimination yields substantial returns by reducing morbidity, mortality, poverty, economic disparities, and health inequities [10]. Furthermore, NTDs are a One Health issue with interdependency between human, animal, and ecosystem health due to the transmission cycles of most NTDs involving animal reservoirs, vectors, and environmental components [11].

**Fig 1:**
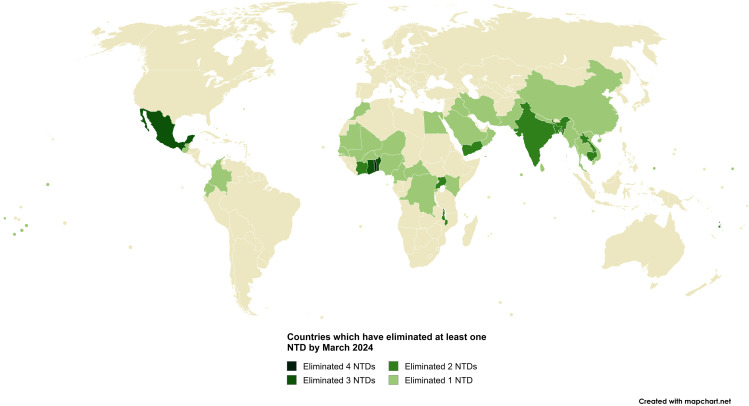
Map of the 50 countries in the world which had each eliminated one or more NTDs by March 2024. Produced on MapChart using the World Map: Microstates base map: www.mapchart.net/detworld.html. Terms of Use available at: www.mapchart.net/terms.html#licensing-maps. References: [12].

It is therefore unsurprising that there are currently global efforts to control and eliminate NTDs in order to reduce human suffering, promote economic development, and progress toward One Global Health. The WHO’s second road map for NTDs details the global vision and goals for the prevention, control, elimination, and eradication of NTDs for 2021–2030 [4]. These goals align with the SDGs and the WHO’s 13th General Programme of Work, and include 1) 100 countries eliminating at least one NTD and 2) the eradication of two NTDs, Guinea worm disease and yaws, by 2030. Encouraging progress has been made towards these targets. By December 2023, 50 countries in the world had already eliminated at least one NTD [12], reaching the halfway mark of the WHO’s goal of 100 countries by 2030 ahead of schedule (Fig 1). By June 2025, this number of countries had risen to 56 with Chad, Jordan, Brazil, Timor-Leste, Guinea, and Papua New Guinea having eliminated their first NTDs [13–18]. By March 2024, the end date of the current review period, eight of the 21 NTDs had been eliminated in at least one country each [12,19]. These eight are: Guinea worm disease (GWD), human African trypanosomiasis gambiense and rhodesiense (gHAT and rHAT, respectively; together known as HAT), lymphatic filariasis (LF), onchocerciasis (ONCHO), dog-transmitted human rabies, trachoma, visceral leishmaniasis (VL), and yaws. The pathogen names, general disease characteristics, and countries where each NTD has been eliminated under specific criteria by March 2024, are listed in Table 1.

**Table 1:** The eight NTDs eliminated (PHP or IoT) in at least one country by March 2024.

Eliminated NTDs	Causative agents, transmission, disease picture	Countries having achieved elimination by March 2024 {total count of countries}	NTD-specific elimination criteria (PHP/IoT)
Guinea worm disease (dracunculiasis)	Caused by *Dracunculus medinensis* worm; transmission via contaminated drinking water; infected persons become incapacitated for weeks as worm painfully emerges through skin [4]	Benin, Burkina Faso, Cameroon, Central African Republic, Côte d’Ivoire, Democratic Republic of the Congo, Ghana, India, Kenya, Mauritania, Niger, Nigeria, Pakistan, Senegal, Togo, Uganda, Yemen {17}	IoT: passing 3 consecutive years without indigenous cases while upholding active surveillance [20,21]
Human African trypanosomiasis gambiense and rhodesiense (sleeping sickness)	Caused by *Trypanosoma brucei gambiense* and *rhodesiense* protozoans; transmission via tsetse fly bite; result in chronic (gHAT) or acute (rHAT) infection, fatal without treatment [4,22]	gHAT: Benin, Côte d’Ivoire, Equatorial Guinea, Ghana, Togo, Uganda {6}; rHAT: Rwanda {1}	PHP: < 1 cases in a population of 10,000 people in all country districts for five consecutive years [23]
Lymphatic filariasis (elephantiasis)	Caused by filarial worm parasites *Wuchereria bancrofti, Brugia malayi, B. timori*; transmission via mosquitoes; results in lymphatic vessel damage, lymphoedema, hydrocoele [4]	Bangladesh, Cambodia, Cook Islands, Egypt, Kiribati, Laos, Malawi, Maldives, Marshall Islands, Niue, Palau, Sri Lanka, Thailand, Togo, Tonga, Vanuatu, Vietnam, Wallis and Futuna, Yemen {19}	PHP: infection prevalence below a measured threshold, minimum morbidity and disability care package must be available [24]
Onchocerciasis (river blindness)	Caused by *Onchocerca volvulus* worm; transmission via repeated blackfly bites; results in sight impairment or permanent blindness [4]	Colombia, Ecuador, Guatemala, Mexico {4}	IoT: 3–5-year post-MDA surveillance, serological tests in children, < 1/1,000 prevalence of PCR-tested infected black flies [25]
Rabies	Caused by rabies lyssavirus and other lyssaviruses; transmission via bites of animals, mainly dogs; results in gradual inflammation and death [4]	Mexico {1}	PHP: effective surveillance system in place, no indigenous cases detected in two consecutive years [26]
Trachoma	Caused by *Chlamydia trachomatis* bacterium; transmission via person-to-person contact or flies; results in sight impairment or blindness [4]	Benin, Cambodia, China, Gambia, Ghana, Iran, Iraq, Laos, Malawi, Mali, Mexico, Morocco, Myanmar, Nepal, Oman, Saudi Arabia, Togo, Vanuatu {18}	PHP: prevalence of trachomatous trichiasis (blinding stage) <1 cases per 1,000 inhabitants; < 5% prevalence of trachomatous inflammation-follicular stage in children within a district [27]
Visceral leishmaniasis (kala azar)	Caused by *Leishmania* protozoans; transmission via female sandfly bites; results in hepatosplenomegaly, anaemia, fatal if untreated [4]	Bangladesh {1}	PHP: cases at <1 per 10,000 inhabitants at (sub)district level (only in Southeast Asia) [28,29]
Yaws (endemic treponematoses)	Caused by *Treponema pallidum* subspecies *pertenue* bacterium; transmission via skin-to-skin contact; results in skin disfiguration [4]	India {1}	IoT: absence of new cases and transmission for three consecutive years with ongoing surveillance [30]

Names and alternative names in brackets are listed for each NTD. Causative agents, modes of transmission, and disease pictures are described. Listed are names and counts of countries having achieved elimination. NTD-specific elimination criteria are detailed according to which type of elimination (PHP or IoT) has been achieved. For acronyms, see S1 Table.

Out of the 50 countries, 46 had achieved NTD elimination specifically as public health problem (PHP) for one or more NTDs. This elimination is defined by the WHO as reducing NTD occurrence to a level where it no longer poses a public health threat, based on clinical symptoms and infection burden [4,31]. Additionally, 22 countries out of the 50 had eliminated one or more NTDs with interruption of transmission (IoT), which the WHO defines as bringing transmission to zero within a region – an achievement that is more challenging. These 46 and 22 countries within the total 50 overlap since different NTDs have been targeted for either PHP or IoT elimination. The WHO’s NTD-specific elimination definitions for the eight NTDs are provided in Table 1. NTD control refers to reduction of incidence, prevalence, morbidity and/or mortality, while eradication, as per the WHO, is the permanent elimination of a disease without the possibility of re-emergence [4].

**Fig 2:**
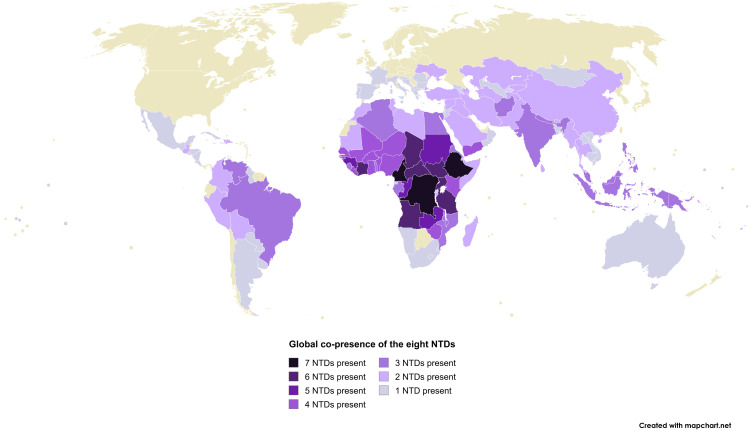
Map of 134 countries with presence of one or more of the eight NTDs by October 2024. gHAT and rHAT are considered separately. With rabies, only dog-mediated human cases are considered. Produced on MapChart using the World Map: Microstates base map: https://www.mapchart.net/detworld.html. Terms of Use available at: www.mapchart.net/terms.html#licensing-maps. References: [32–44].

As seen in Fig 2, 134 countries, and possibly more, remain affected by one or more of the eight NTDs. When considering the remaining 13 NTDs, this count of countries is possibly even higher. Lessons from elimination successes and failures can offer valuable insights to these countries where NTDs are still endemic. Thus, our aim is to examine the elimination approaches used in the 50 countries that have successfully eliminated at least one NTD, as well as their failed elimination attempts, and synthesise a blueprint for NTD elimination.

## Methods

### Literature search strategies and selection criteria

For each of the 50 countries, we conducted literature searches of publications detailing aspects of their NTD control and elimination strategies. Searches of published literature were conducted primarily on PubMed and secondarily on infoNTD databases between the 5th of February and 22nd of May 2024. Searches were conducted individually for each eliminated NTD, using the search terms and strategies in Table 2. The search strategy format was initially piloted on PubMed, using lymphatic filariasis, the first NTD for which searches were conducted, to assess whether the strategy retrieved relevant results. The relevancy of retrieved publications was assessed by the total number of results retrieved and scanning of publication titles to inspect whether most met the designated inclusion criteria. The strategy was adjusted accordingly to improve results. Search strategies only contained terminology in English. No filters were used for language or publication dates in either database. To ensure all relevant publications were identified and included, second searches and screening using the same search terms and inclusion criteria were conducted on PubMed by a second independent reviewer.

**Table 2:** PubMed search strategies used for the eight NTDs eliminated in at least one country by March 2024.

NTDs	PubMed search strategies	Number of PubMed results from searches
GWD	(Guinea worm disease OR dracunculiasis) AND (control OR elimination OR eradication OR surveillance) AND (Pakistan OR India OR Yemen OR Kenya OR Uganda OR Cameroon OR Nigeria OR Niger OR Benin OR Togo OR Ghana OR Côte d’Ivoire OR Senegal OR Mauritania OR Burkina Faso OR Democratic Republic of the Congo OR DRC OR Central African Republic)	343
HAT	(Human African trypanosomiasis gambiense OR Human African trypanosomiasis rhodesiense OR sleeping sickness) AND elimination AND (Uganda OR Rwanda OR Equatorial Guinea OR Benin OR Togo OR Ghana OR Côte d’Ivoire)	88
LF	(lymphatic filariasis OR elephantiasis) AND (elimination OR control OR eradication) AND (Palau OR Marshall Islands OR Kiribati OR Wallis Futuna OR Vanuatu OR Tonga OR Niue OR Cook Islands OR Thailand OR Cambodia OR Vietnam OR Viet Nam OR Laos OR Lao People’s Democratic Republic OR Bangladesh OR Sri Lanka OR Maldives OR Egypt OR Yemen OR Malawi OR Togo OR Oceania OR Pacific)	385
ONCHO	(Onchocerciasis OR river blindness) AND elimination AND (Mexico OR Guatemala OR Ecuador OR Colombia)	66
RABIES	Rabies AND elimination AND Mexico	15
TRA	Trachoma AND elimination AND (Vanuatu OR Mexico OR Mali OR Gambia OR Ghana OR Togo OR Benin OR Malawi OR Morocco OR Saudi Arabia OR Iraq OR Iran OR Oman OR China OR Laos OR Lao People’s Democratic Republic OR Nepal OR Myanmar OR Burma OR Cambodia)	166
VL	(Visceral leishmaniasis OR kala azar) AND elimination AND Bangladesh	101
YAWS	(Yaws OR endemic treponematoses) AND elimination AND India	7

infoNTD searches were equivalent except for being divided into separate searches for NTD common and scientific names. The most effective search strategy to obtain a reasonable number of relevant results was of the following format: (*scientific name of NTD* OR *common name of NTD*) AND elimination AND (*list of countries and/or regions where NTD in question has been eliminated, with alternative names of some countries*). NTD abbreviations: GWD, Guinea worm disease; HAT, human African trypanosomiasis; LF, lymphatic filariasis; ONCHO, onchocerciasis; TRA, trachoma; VL, visceral leishmaniasis.

Publications to be included were required to contain information on (1) the elimination of one of the eight NTDs, (2) focus on countries or regions where elimination has been achieved, and (3) detail the elimination efforts that have led to a validated or verified elimination. Publications were excluded if they were not available in English, Spanish, or French, or if the information they provided had already been sourced from another publication or source. Beyond published literature, we searched for additional information relevant to the elimination efforts from the WHO Institutional Repository for Information Sharing (IRIS) digital library [45], the WHO Global Health Observatory [46], multi-year NTD elimination master plans of several African countries available and examined on the website of the WHO Regional Office for Africa’s Expanded Special Project for Elimination of Neglected Tropical Diseases (ESPEN) [47], and from the WHO website news releases and fact sheets. These sources of information were selected based on the organisational authority, credibility and relevance of the World Health Organization. Where necessary, the following other grey literature sources were used to obtain programmatic data: Act to End NTDs West, the Carter Center, Ecuador’s Ministry of Public Health, the Indian Government’s Ministry of Health and Family Welfare, Saudi Arabia’s Ministry of Health, the International Agency for the Prevention of Blindness, International Coalition for Trachoma Control, Liverpool School of Tropical Medicine, the Pan American Health Organization and the United States Agency for International Development. These sources were deemed reliable due to their organisational credibility, reputability, and relevance. Where possible, reliability of programmatic data provided by these sources was checked by comparing to data available from published literature. Inclusion criteria for grey literature were as for published literature: websites, documents and reports of the above organisations were included as sources if they contained detailed information on the components of elimination efforts and programmes for one of the eight NTDs in a country or region where elimination has been validated or verified. Relevant data were located either via webpage navigation or via ChatGPT 4.0 Scholar. ChatGPT was used to discover online sources and relevant publications by prompting with requests to localise specific information and provide citations: e.g., “What year did Rwanda’s elimination programme for human African trypanosomiasis rhodesiense begin? Provide in-text citations.” No information or data was directly extracted from ChatGPT; citations provided by ChatGPT were used to directly navigate to the publication, website, or other source to examine and extract relevant information.

### Data extraction, synthesis and visualisation

**Fig 3:**
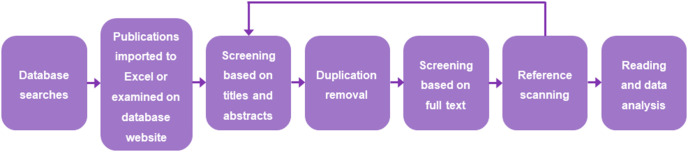
Flow chart depicting the literature search, publication inclusion and reading process. PubMed search results were downloaded as Excel files, while infoNTD results were examined on website. Reference scanning refers to discovery of relevant publications from the references of publications already included from literature searches. This figure was produced in PowerPoint.

Detailed information was extracted from the literature and additional sources. This included: (1) types of elimination achieved (PHP/IoT), (2) start and validation/verification years of elimination programmes, (3) elimination interventions and strategies implemented, (4) programme structuring and degree of integration with other programmes and into the health system, (5) NTD elimination programme organisers and leaders, (6) partners involved in supporting elimination and types of support they provided, and (7) historical NTD control programme characteristics. NTD endemicity, or presence when endemicity data was not available, and country status information was used to produce Figs 1 and 2 using MapChart (mapchart.net). The identified partners contributing to elimination efforts were summarised in a bubble graph produced in PowerPoint. Synthesised summaries of elimination programme durations, integration levels, elimination strategies, and partner support types were generated using GraphPad Prism 10.2.2. The NTD elimination road map was produced in PowerPoint. The process of reviewing to synthesis is summarised Fig 3. Although a systematic search was conducted, this was not a formal systematic review or meta-analysis. Therefore, record counts were not tracked, and the flow diagram illustrates the process conceptually rather than quantitatively.

## Results

### Durations of elimination processes

**Fig 4:**
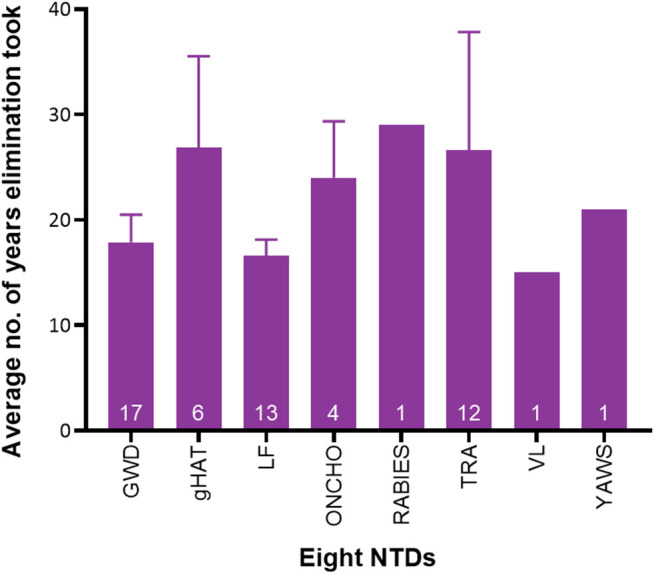
Average duration of the NTD elimination process for the eight NTDs whose elimination has been achieved in at least one country. Sample sizes, i.e., number of countries with elimination programmes for which information was found, are denoted inside bars for each set of data. Data sources: GWD: [19,48–71]; HAT: [48–50,72–75]; LF: [50,76–93]; Onchocerciasis: [94–99]; Rabies: [100]; Trachoma: [19,50,101–115]; VL: [116,117]; Yaws: [118,119].

Based on launch years of national elimination programmes and efforts, and years in which eliminations were validated or verified by the WHO in each country, the average elimination process durations were calculated and are shown in Fig 4. The durations for rabies, VL and yaws reflect single-country eliminations, while 95% confidence intervals are provided for NTDs eliminated in multiple countries. Elimination process durations range from 15 to 29 years, with an overall average of approximately 22 years. Notably, launch year data were unavailable for LF elimination efforts in six countries (Kiribati, Laos, Maldives, Marshall Islands, Palau, Wallis and Futuna), trachoma in six countries (Cambodia, Iran, Laos, Malawi, Saudi Arabia and Vanuatu), and rHAT in Rwanda, so these were excluded from Fig 4.

### NTD control strategies employed in elimination programmes

#### Grouping of interventions into strategy categories.

The common elimination interventions and strategies which were employed across the eight NTDs and consistently appeared in literature and other sources were recorded. Interventions refer to individual actions which accelerate elimination, while strategies encompass a range of interventions. The common interventions are grouped into eight strategy categories detailed in Table 3. Four of these strategies are preventive chemotherapy (PC), water, sanitation and hygiene (WASH), vector control, and veterinary public health, as recommended by the WHO for NTD prevention, control, elimination, and eradication [120]. We grouped the other reported interventions into case detection and disease monitoring, case management, health system capacity building and strengthening, and community engagement and education (Table 3). Additionally, the WHO-endorsed SAFE strategy (Surgery, Antibiotics, Facial cleanliness and Environmental improvements) employed for trachoma [4,103] has been divided into its four components. These components have been allocated across three of the eight strategies used in this review (Table 3).

**Table 3:** The eight NTD elimination strategies as defined in this report, alongside their definitions and individual interventions included in each strategy.

NTD elimination strategies	Definitions of strategies	Interventions included in strategies	
Case detection and disease monitoring	Detection and diagnosis of individual cases, and monitoring disease incidence, prevalence, and geographical spread	• Prevalence mapping (questionnaires, surveying, historical records) • Screenings • Surveillance (active and passive, post-MDA)	• Entomological surveying • Active case searches • Investigating rumoured cases • Case reporting and recording
Case management	Action taken to manage individual diagnosed cases, and prevent onwards transmission, morbidity, disability, and death	• Appropriate medical attention • Component S of SAFE strategy (Surgery) • Case containment • Case containment centres	• Morbidity management and disability prevention (MMDP) (including hydrocoele surgeries, morbidity management kits) • PEP for dog-bitten people
Health system capacity building and strengthening	Development and enhancement of country’s ability to conduct elimination efforts, and strengthening elimination programmes	• Training of medical professionals/health workers/ field workers/volunteers • Monitoring and evaluation of elimination programme performance • KAP surveys • Innovative employment of technologies (e.g., SMS systems)	• Replacement of poorly performing health workers or volunteers • Research (operational, clinical) • Integration (with other interventions, elimination programmes, or into primary health care system) • Advocacy • Involvement of local women in elimination efforts • Motivation incentives for volunteers and health workers
Community engagement and education	Involvement of affected communities in elimination efforts, and enhancing their knowledge of NTDs and their elimination	• Social mobilisation and sensitisation • Health education • MDA awareness campaigns • Advertisement campaigns	• IEC and COMBI campaigns • Education on correct use of water filters • Education on safety of interventions (e.g., ABATE) • Cash rewards for case reporting
Preventive chemotherapy	Mass drug administration to eligible members of communities in endemic or at-risk areas to prevent and treat cases [4,121], accompanied by surveying	• Mass drug administration (MDA) • Component A of SAFE strategy (Antibiotics) • Preventive treatment of infected person’s contacts	• MDA-related surveys (coverage and impact surveys, TAS, stop- and pre-stop-MDA surveys, post-MDA surveys)
WASH (water, sanitation and hygiene)	Ensuring and improving access to safe water, sanitation and hygiene [4,122]	• Improving safe water access/supply/ provision • Building and rehabilitating wells and other safe water sources	• Use of water filters • Advocacy with water organisations • Components F and E of SAFE strategy (Facial cleanliness, Environmental improvements)
Vector control	Action to diminish vector population densities to reduce NTD transmission [123]	• Stagnant water source treatments with larvicide (ABATE) • Insect traps (e.g., Tiny Targets)	• Vector control as part of malaria/dengue programmes (LLINs) • Integrated vector management (IVM)
Veterinary public health	Diminishing animal reservoir to reduce transmission to humans [124]	• Animal trypanosomiasis diagnosis and treatment	• Annual mass dog vaccination campaigns • Cat and dog sterilisation

References within definition boxes are to the model WHO strategies, which may be slightly modified here for the purposes of this review. Only the interventions listed in this table are considered in this report. For acronyms and abbreviations used, see S1 Table. References: [48–60,62,64,66–68,73,76–91,94–97,99–113,116–118,125–179].

#### Programmatic employment of elimination strategies across NTDs.

**Fig 5:**
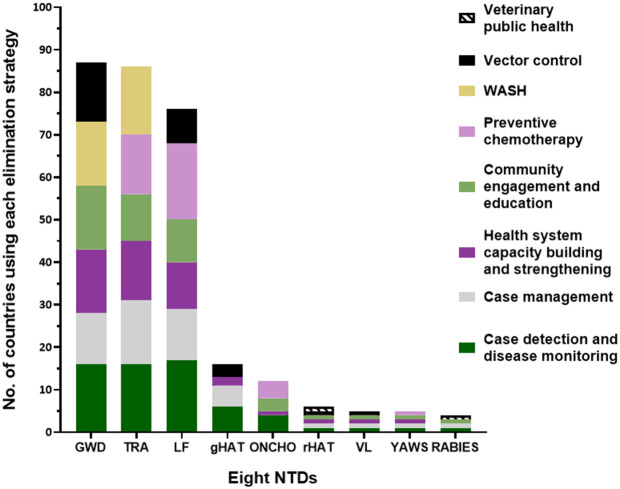
Elimination strategies employed across the eight NTDs in the 50 countries having achieved elimination. On the x-axis are the eight NTDs, with gHAT and rHAT presented separately. On the y-axis are numbers of countries which have employed each strategy per NTD. Each section of a stacked bar therefore represents the number of countries whose elimination programmes or efforts have employed the strategy for the NTD in question. A point system was used to generate this graph: each strategy was given a point per NTD each time at least one intervention included in this strategy was used in an elimination programme or line of efforts for the NTD in question. References: [48–60,62,64,66–68,73,76–91,94–97,99–113,116–118,125–182].

The review showed that different strategy combinations were used for the different NTDs (Fig 5). While some strategies such as case management were used across most NTDs, others such as veterinary public health were related to the mode of transmission. Detailed information on the employment and components of the eight strategies is provided in S2 Table.

Case detection and disease monitoring was the most frequently used strategy, employed in 63 programmes across all eight NTDs in the 50 countries. The lack of concordance between the number of programmes and countries is a result of some countries having multiple NTD programmes. Case management was used in 48 programmes, for all NTDs excluding onchocerciasis, while health system capacity building and strengthening was implemented in 46 programmes for all NTDs except rabies. Community engagement and education was utilised in 43 programmes, excluding gHAT. PC was employed in 37 programmes targeting trachoma, LF, onchocerciasis, and yaws. To date, no country has been verified for the elimination of the fifth PC NTD, schistosomiasis. WASH has been applied in 31 programmes against GWD and trachoma. Vector control targeting Guinea worm larvae-harbouring copepods, mosquitoes, tsetse flies, or sandflies was utilised in 27 programmes for GWD, LF, HAT, and VL. Veterinary public health approaches, such as vaccination of dogs which are rabies reservoirs, and diagnosis and treatment of trypanosome-infected livestock, have been used in two elimination programmes, those of Mexico for rabies and Rwanda for rHAT [100,146,160,162].

### Elimination programme structure and integration

Iran and Saudi Arabia were the only countries to eliminate an NTD (trachoma) without dedicated elimination programmes or specific efforts. Instead, they relied on primary health care (PHC) services and the general implementation of the WASH strategy [105,125]. In contrast, all other countries ran dedicated single or multi-disease-specific NTD elimination programmes or conducted dedicated efforts, even if not formally organised as official national elimination programmes [48–60,62,64,66–68,73,76–91,94–97,99–113,116–118,125–179].

**Fig 6:**
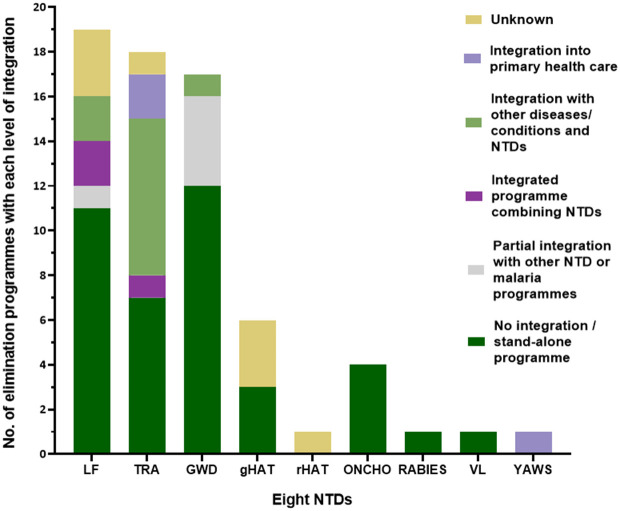
Levels of integration in elimination of the eight NTDs. On the x-axis are the eight NTDs, with gHAT and rHAT presented separately. On the y-axis are numbers of countries which have eliminated each NTD. Each section of a stacked bar represents the number of countries that have eliminated an NTD using a programme or line of effort with the colour-denoted level of integration. When sources discussed disease-specific programmes without mention of integration into primary healthcare or shared efforts, these programmes have been assumed to be stand-alone.

Fig 6 shows the varied levels of integration of NTD control and elimination efforts into programmes targeting multiple NTDs or other diseases, or the mainstreaming of elimination programmes into national health systems and activities. Overall, 39 elimination programmes across NTDs were non-integrated stand-alone programmes targeting one NTD only at a time. Five programmes across LF and GWD had partial integration with other NTDs or malaria. These programmes had stand-alone structures yet integrated some interventions, such as vector control, MDA, or active case searches, with other NTDs or malaria. Three programmes across LF and trachoma were fully integrated NTD programmes, meaning they combined efforts against two or more NTDs. 10 integrated programmes across LF, trachoma and GWD targeted one or more NTDs along with other diseases and conditions. Some of these programmes employed cross-cutting strategies like WASH, and seven targeted trachoma through general agendas for eye health, blindness prevention, or communicable diseases [101,105,107,111,113,164,172,183,184]. India had a dedicated yaws programme which was delivered through existing health systems in place [118,178]. For eight programmes and lines of effort across LF, trachoma and HAT, information on the level of integration was not found or was unclear.

### Operational aspects

#### Organisers of elimination programmes and efforts.

We found that both local governments and international development partners have been involved in facilitating NTD elimination. In most countries, national Ministries of Health (MoH) and similar institutions have been organisers of elimination efforts, and particularly in African countries have developed and subsequently implemented master plans for NTD control and elimination. National universities, institutes, councils, and government ministries outside of the MoHs have been involved in supporting the delivery of elimination programmes and efforts. In Iraq and Nepal, for example, Ministries of Health collaborated with respective Ministries of Education to organise trachoma screenings in school-aged children (Iraq) and integrate trachoma modules into the school curriculum (Nepal) [106,112].

#### Partners supporting NTD elimination.

**Fig 7:**
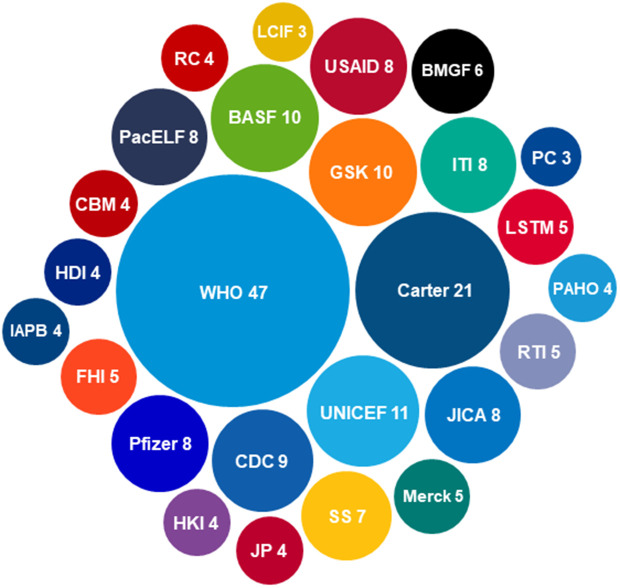
Partners supporting three or more NTD elimination programmes and efforts across the eight NTDs. For definitions of partner name abbreviations and acronyms, see Table 4. Size of each bubble, and the number shown after the partner name/acronym, correspond to number of individual elimination programmes and efforts supported by each partner. A point system was used to generate the graph: a partner was given a point for each individual NTD elimination programme or line of efforts it has supported. Each partner could only be given one point for each individual programme it supported: extent or type of support was not considered. Data used to generate this graph was gathered from 63 NTD-country combinations for which information was found. References: [48–56,59–66,69,72,75–81,84,86–89,91,94,96–98,101–104,106,108–113,116,117,126,129,133–137,140,145,146,148,152,157,158,162,168–170,172–174,177,178,184,187–203].

International organisations have supported several elimination programmes. In Fig 7 and Table 4, we summarise the partners which supported three or more elimination programmes across the eight NTDs eliminated in at least one country to date. Partners that have supported one or two elimination programmes are listed in S3 Table. The WHO has supported the largest number of countries, 47, with the Carter Center and UNICEF supporting 21 and 11 countries, respectively. In addition to partners shown in Fig 7, Uniting to Combat NTDs, a global NTD advocacy and resource mobilisation organisation, has contributed to NTD elimination efforts by building political willpower and mobilising financial resources through the 2012 London Declaration on NTDs [185], and its successor, the 2022 Kigali Declaration [186].

**Table 4:** Full names of partners having supported three or more NTD elimination programmes (shown in Fig 7).

Partner name abbreviation/ acronym	Partner name definition
BMGF	Bill and Melinda Gates Foundation
Carter	the Carter Center
CBM	Christian Blind Mission
CDC	Centers for Disease Control and Prevention (US)
FHI	FHI 360
GSK	GlaxoSmithKline
HDI	Health & Development International
HKI	Helen Keller International
IAPB	the International Agency for the Prevention of Blindness
ITI	International Trachoma Initiative
JICA	Japan International Cooperation Agency
JP	Japanese government
LCIF	Lions Clubs International Foundation
LSTM	Liverpool School of Tropical Medicine
PacELF	the Pacific Programme for the Elimination of Lymphatic Filariasis
PAHO	Pan American Health Organization
PC	Peace Corps
RC	Red Cross
RTI	RTI International
SS	Sightsavers
UNICEF	the United Nations International Children’s Emergency Fund
USAID	United States Agency for International Development
WHO	World Health Organization

#### Types of support provided by partners.

**Fig 8:**
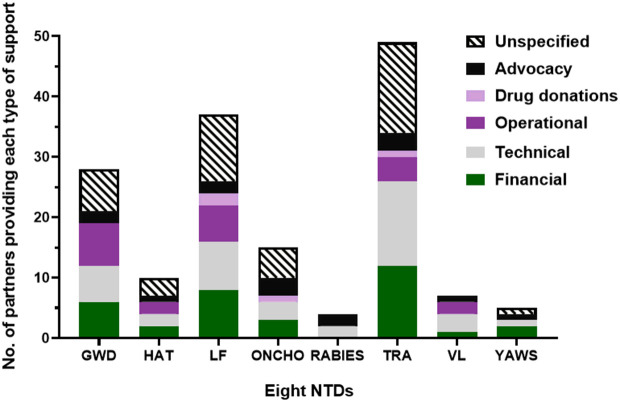
Types of support provided by partners contributing to the elimination of the eight NTDs at any point or for any duration of time during the elimination process. On the x-axis are the eight NTDs. On the y-axis are numbers of partners which have provided each type of support. Each section of a stacked bar represents the number of partners that have provided the colour-designated type of support to aid elimination of each NTD. A point system was used to generate this graph: a partner was given a point for each type of support per NTD when it had provided this for at least one relevant programme.

The types of support provided by partners who supported at least three programmes (see Fig 7) are summarised in Fig 8. Details and examples of support provided by these partners are available in S4a and S4b Table. Types of support include financial assistance to cover the partial or whole costs of running elimination programmes, and delivering interventions such as MDA. Technical support involved control strategy and NTD master plan development, protocols, and expert guidance. Financial and technical support also included capacity building in the form of staff training and infrastructure strengthening. Operational support towards programme implementation included activities such as procurement of drugs and diagnostic kits, and assistance with carrying out of interventions in the field. PC drugs were donated by pharmaceutical companies, with the donations being managed by non-pharmaceutical organisations including the WHO. Advocacy efforts promoted elimination, mobilised resources, and facilitated regional initiatives. Unspecified support refers to cases where the type of assistance was not detailed. Most of the NTD elimination programmes received most forms of support to varying levels, except for rabies possibly due to it not being a PC NTD.

### Challenges to elimination

In addition to the cornerstones of success detailed above, our review also included investigating historical failures in elimination. These are summarised in Table 5. Analyses of 11 failed elimination efforts revealed that although the programmes failed to eliminate the NTDs, seven of these did succeed in reducing infection levels (Table 5). Also, efforts against HAT (in several African countries) [142], VL (Bangladesh) [204], and yaws (India) [118] came close to elimination. Nonetheless, case resurgences, insufficient case decreases, and delayed progress hindered elimination. Other reasons for elimination failure were resource limitation, insufficient intensity in carrying out interventions, lack of effective interventions, socio-political changes, deprioritisation, as well as instability and conflicts. Notable is that these reasons for failure are intertwined. S5 Table provides more detailed aspects of some of the historical failed programmes.

**Table 5:** Characteristics of historical NTD control and elimination programmes, campaigns, and efforts which failed to achieve WHO-validated or -verified NTD elimination status.

NTD	Country	Historical control/elimination efforts	Lowered case numbers?	End point	Causes of resurgence and/or delay in elimination					Ref.
					Lack of funds/capacity/resources/staff/time	Insufficient intensity of interventions	Lack of effective interventions	Socio-political changes, instability, conflicts	Lack of prioritisation/ de-prioritisation	
HAT	Uganda	Wide-scale screening and treatment campaigns	Yes	Resurgence				✓	✓	[142,205,206]
HAT	Equatorial Guinea	Screenings, requirement of health passport for travelling, proof of screening participation for employment	Yes	Resurgence	✓			✓	✓	[73]
LF	Egypt	Endemicity surveys, selective DEC case treatment, intensive vector control	Yes	Resurgence	✓	✓				[79]
LF	Maldives	Prevalence surveys, selective DEC case treatment, vector control	Yes	Unknown		✓	✓			[154]
ONCHO	Mexico	Nodulectomies, selective DEC case treatment, sporadic vector control (DDT)	Unknown	Unknown		✓	✓			[96,97,159]
ONCHO	Ecuador	Systematic nodulectomies	No	Unknown		✓	✓			[99]
ONCHO	Guatemala	Nodulectomies, health worker brigades, sporadic treatment with DEC, some vector control	Unknown	Unknown		✓	✓			[95,98]
RABIES	Mexico	Wide-scale dog vaccinations 1970s-1980s	No	Cases not sufficiently lowered		✓			✓	[160,207]
TRA	Mexico	Case surveillance, hygiene promotion, use of antiseptics, antibiotics, surgeries, building of hospitals and health centres, health brigades did case detection and reporting, health examinations	Yes	Unknown	✓	✓			✓	[96,108]
Malaria (VL)	Bangladesh	Collateral sandfly control via malaria eradication programme 1950s-1970s (IRS with DDT)	Yes	Resurgence		✓	✓			[176,177,204]
YAWS	India	Arsenicals used 1935–1946. In 1952, a control programme used penicillin for treatment	Yes	Resurgence/ re-emergence	✓	✓			✓	[118,178]

Interventions conducted against NTDs in listed countries are summarised. The endpoint results, whether case numbers were decreased, and overall reasons for programme failure are denoted. For acronyms, see S1 Table. NTD abbreviations: HAT, human African trypanosomiasis; LF, lymphatic filariasis; ONCHO, onchocerciasis; TRA, trachoma; VL, visceral leishmaniasis.

### Road map for NTD elimination

**Fig 9:**
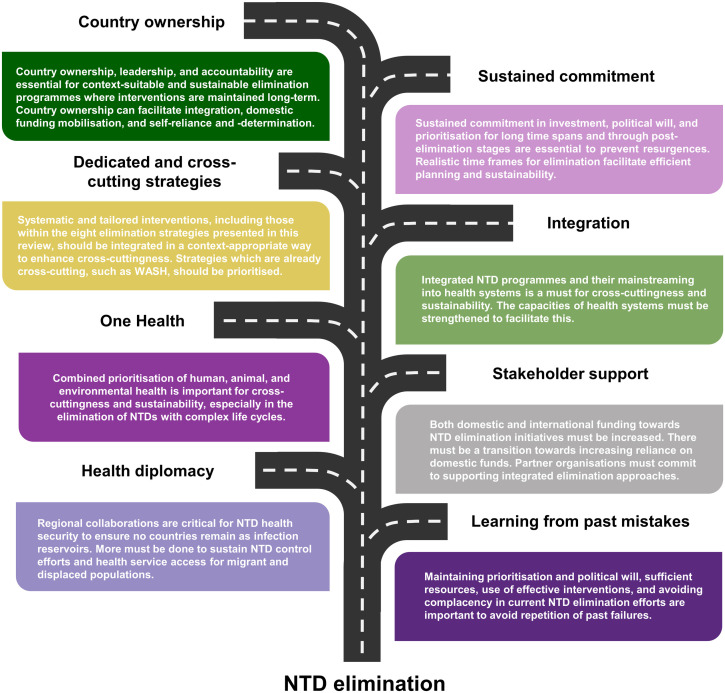
Road map for NTD elimination based on the findings of this review. This figure was produced in PowerPoint.

Based on the findings of this review, a road map detailing the blueprint for NTD elimination has been compiled (Fig 9). For ongoing and future NTD elimination programmes to be successful, they must incorporate country ownership, sustained commitment, dedicated and cross-cutting strategies, integration, One Health approaches, stakeholder support, health diplomacy, and lessons from historical failures.

## Discussion

Neglected tropical diseases are compromising progress towards targets of several poverty, health, and education SDGs [4,8,9]. Thus, the WHO has set a target for 100 countries to eliminate at least one NTD by 2030 [4]. By March 2024, 50 countries had achieved this goal, and by June 2025, this number had risen to 56 countries [18]. To meet the WHO’s target, 44 more countries must eliminate an NTD by 2030, highlighting the need to both accelerate and intensify elimination efforts. In this work, we analysed the features of NTD programmes in the 50 countries associated with successful elimination by March 2024 and their historical elimination failures to produce a blueprint that can inform programmes in other countries.

A significant feature of NTD elimination across all 50 countries was the time taken to reach elimination. The average duration of successful NTD elimination processes was over two decades [19,48–119], underscoring the need for sustained time commitment, investment, systematic interventions, and political will and prioritisation. This is underlined by experiences from historical elimination efforts in, for example, Uganda and India, where lack of long-term investment and sustained control efforts led to resurgences of HAT and yaws, respectively [118,142,178,205,206]. The maintenance of realistic time frames for long elimination processes can help ensure efficient planning, resource allocation and sustained motivation. It also helps reduce the possibility of donor hesitancy or withdrawal [208]. Long time spans also require NTD programmes to be resilient to endure potential disruptions such as conflict, economic and political shifts, or changing/sudden pressures on health systems such as those seen during the COVID-19 pandemic [209]. All of these aspects are particularly important for the last mile efforts against NTDs like GWD and yaws, both targeted for eradication [4,210].

Failed historical NTD programmes and efforts provide useful lessons to apply in today’s efforts to eliminate more NTDs in more countries. Firstly, there must be no room for complacency to stand in the way of elimination. Political prioritisation of elimination must be upheld throughout, and efforts sustained all the way to elimination and beyond to prevent NTD pre- or post-elimination resurgence. Since country elimination statuses are reversible, it is imperative that countries sustain post-elimination interventions such as surveillance to not lose or undo progress, as recommended by the WHO [31,208]. Countries should receive standardised guidance on post-elimination activities [108], and elimination efforts must be supported with adequate resources and efficient interventions over long timeframes. The existence and development of effective interventions, drugs, supplies, and technologies to beat NTDs is integral.

Another key finding was that countries successful in NTD elimination had ownership of their programmes and dedicated elimination initiatives [48–56,59–66,69,72,75–81,84,86–89,91,94,96–98,101–104,106,108–113,116,117,126,129,133–137,140,145,146,148,152,157,158,162,168–170,172–174,177,178,184,187–202]. For example, the Ugandan government’s strong leadership in efforts against GWD [59], Niue’s government’s stable support for LF elimination [81], and the Chinese government’s commitment to trachoma prioritisation [167] have been cornerstones of these countries’ successes in eliminating these diseases. Indeed, country ownership is essential for context-suitable elimination structuring, and its importance is emphasised in the Kigali Declaration of 2022 [9]. In the WHO’s NTD road map for 2021–2030, Pillar 3 focuses on changing operating models and culture to facilitate country ownership of NTD control programmes [4]. This is a critical step for community buy-in and uptake as well as long-term sustainability as highlighted before. Government commitment to and accountability over elimination efforts can ensure their maintenance pre- and post-elimination and facilitate NTD programme integration into national health systems [211]. Domestic political will can allow domestic funding mobilisation and funnelling to elimination efforts [211], thereby enhancing country self-determination and reducing reliance on external funding.

Support of various partners external to national MoHs has also been key in NTD elimination. Support provided by partners has included financial, technical and operational support, capacity building, drug donations, and advocacy [48–56,59–66,69,72,75–81,84,86–89,91,94,96–98,101–104,106,108–113,116,117,126,129,133–137,140,145,146,148,152,157,158,162,168–170,172–174,177,178,184,187–202]. Each type of support has mostly been provided by more than one partner, ensuring availability of complementary and interdisciplinary support. This is crucial for a One Health approach which harnesses transdisciplinary collaboration between stakeholders [11]. Cooperation between partners, as practised by, e.g., WHO and ITI with pharmaceutical companies (S4a and S4b Table), is also important to ensure resource optimisation. As suggested by the heavy involvement of partners, successful elimination has at least in some cases been dependent on external financial support [50,208,212], particularly as the eight NTDs mostly affect lower-income countries [213]. This has been the case for, e.g., Togo’s HAT and LF elimination programmes which relied entirely on funding from partners (S4a and S4b Table) [50]. Other countries, on the other hand, have entirely or mostly funded their elimination efforts domestically; examples include the Mexican government’s onchocerciasis MDA programme [97] and India’s yaws programme [178].

To facilitate continued global progress against NTDs, both domestic and international funding of NTD elimination efforts should be increased. Cost estimates for some NTD elimination programmes are available: Uganda’s GWD elimination programme was estimated to cost 5.6 million US dollars [59], while Mexico’s onchocerciasis programme cost 57.5 million US dollars [96]. Significant investment is therefore needed, particularly as last-mile elimination interventions may have reduced cost-effectiveness or higher costs [20,214].

Despite the benefits and essentiality of partnerships for NTD control and elimination, heavy reliance on external funding is not without limitations. Partner support may compromise domestic leadership and self-determination, and be unpredictable and limited in duration or be withdrawn completely in the face of conflicts, political instability, geopolitical changes, or shifts in donor country political priorities [107,215]. This has been seen most recently with the foreign aid cuts by the United States and United Kingdom [216,217]. Over-reliance on external funding can thus render NTD programmes fragile and undermine their resilience [218]. Heavy partner involvement may also hinder integration as, for varied reasons, donors may prefer to fund stand-alone elimination programmes over integrated ones [50,168,208,215,219,220]. This can also compromise equal distribution of resources across NTDs [214]. Countries must therefore organise alternative funding routes, generate national budgets for elimination plans, strive for developing and prioritising domestic funding channels [221], and explore innovative funding mechanisms [222]. Collaboration is required from countries and partners to orchestrate a gradual transition in funding NTD elimination increasingly from domestic funds and less from international partners, all the while carefully maintaining capacity that has been built [223]. Partner organisations must ensure their operations support integrated NTD elimination models and direct investments towards integrated, cross-cutting approaches, particularly prioritising One Health [11,223].

This review identified eight key strategies which have been employed in combinations across NTDs to achieve elimination (Fig 5). We recommend that, to maximise impact and use of resources, countries integrate these strategies in a co-endemicity-dependent and context-appropriate way to generate cross-cutting approaches to progress towards the WHO-set 2030 goals [219]. For example, various African and South American countries affected by LF, onchocerciasis, and trachoma might employ integrated PC to target these diseases together [39,42,43]. Some of the eight strategies identified are already cross-cutting. For example, health system capacity building and strengthening, whether in the form of infrastructure, expertise, or facilities, can generally support the fight against NTDs and other diseases, and over time diminish the need for partner support [224]. The WASH strategy, providing access to safe water, sanitation and hygiene, can contribute to improving population health by reducing transmission of NTDs and waterborne and communicable diseases which thrive in poor hygiene and sanitation conditions [122,225]. Safe water access is also necessary for the management of NTD morbidities. Integrated vector control strategies, on the other hand, can simultaneously target insects, snails, and water fleas, which act as transmitting vectors or intermediate hosts for at least 12 different NTDs [4,123]. Veterinary public health promotes animal health in support of human health to control zoonotic NTDs like rabies.

These cross-cutting strategies align with the One Health approach which is crucial for sustainable NTD control and elimination as it considers the interdependency between human, animal and ecosystem health [11,226]. Indeed, NTD control and elimination strategies are ultimately not sustainable if they only account for the human components of NTD transmission cycles. For example, effective rabies control relies on systematically vaccinating dogs as this is the most sustainable and cost-effective way to prevent rabies deaths in humans [11]. A One Health systems thinking approach also allows for consideration of various underlying factors and root causes maintaining and exacerbating NTD burdens, including poverty and economic barriers, environmental factors, social inequalities, and climate change [11]. Consideration of these factors will allow for more effective dismantling of NTD burdens by tackling their upstream drivers [11,227]. Although a cross-cutting One Health framework is vital for long-term, sustainable control of all NTDs, it is particularly necessary for NTDs like schistosomiasis that have complex life cycles involving, e.g., several hosts and environmental contamination. Indeed, biological characteristics of NTDs may ease or hinder elimination processes. The to-date successful eliminations of, e.g., GWD, gHAT, LF, onchocerciasis, and trachoma in four or more countries each have likely been partly facilitated by their biological characteristics favourable to elimination. These characteristics include humans being principal definitive hosts or reservoir, limited spill-over from animal reservoirs in the case of GWD, and lack of a vector in the case of trachoma.

Most successful elimination efforts targeted a single NTD at a time through dedicated programmes, while fewer integrated NTD efforts to various levels in their programmes. Crucially, the WHO’s 2021–2030 NTD road map encourages a shift from these stand-alone programmes towards integrated ones [4]. The transition from parallel vertical programmes towards programmes targeting multiple NTDs and/or other diseases would foster the employment of cross-cutting approaches which offer several benefits, including greater cost-effectiveness, resource optimisation, and minimising duplication of effort, all key considerations particularly in low-resource settings [219,228]. Thus, far some successful elimination programmes, including those of several African and Asian countries for trachoma [101,111,164,172], had integrated programmes targeting NTDs alongside other diseases or conditions such as malaria or causes of blindness. Togo, the country which has thus far eliminated the highest number of NTDs per country (four), has likely been so successful in NTD elimination thanks to its adoption of integrated activities against NTDs like LF and trachoma [50].

Mainstreaming NTD control and elimination into the health system, particularly integration into the PHC, has the potential to accelerate efforts. Already 16 African countries have developed NTD master plans which target various NTDs and identify integration opportunities [48–50,54,56,61,65,69,70,72,75,130,145,168,183,184,229]. Moving away from fragmented and siloed budgeting and organisation to integrated models also allows for NTD prioritisation to be fitted within broader health goals within a country [230]. Cross-sectoral integration of NTD elimination with poverty reduction or nutrition programmes would align with a One Health approach to creating sustainable changes via addressing underlying determinants of NTD burdens [215,227,231]. Integration also facilitates interdisciplinary collaboration between different governmental departments and ministries; this has been done by Iraq, Morocco and Nepal in their successful trachoma elimination programmes [106,110,112]. To support integration, the WHO has developed a strategic framework for integration of skin NTDs [232], and in 2022, 11 countries endemic for at least two skin NTDs had already implemented integrated strategies [12]. Similar approaches could be applied to ‘ocular NTDs’ via general eye health programmes [233]. Ultimately, governments should aim to fully integrate NTD interventions into health systems.

A critical challenge to integrating NTD efforts into PHC is the current weakness of health systems in many NTD-endemic regions [219]. Without a strong healthcare foundation, integrating NTD efforts into PHC will not be feasible or sustainable. A prime historical example of this is India’s failed yaws elimination efforts, which partly relied on general health services that were non-existent in some areas [178] (S5 Table). Weak health systems also hinder the durability of programmes and maintain reliance on external support if NTD programmes and health victories cannot be sustained by the existing health sector. In areas where PHC is underdeveloped or non-existent, strengthening health infrastructure, resources, and workforce capacity first is essential [219,234]. National governments and international stakeholders must collaborate to engage the various elements required to create strong health systems, including robust governance and leadership, political commitment to action, national investment and accountability, prioritisation of quality care and health outcomes [235], and sufficient trained workforce [236].

The global community’s support is needed to prevent socio-political instability and conflicts which disrupt and delay health initiatives including NTD control. Disruptions to NTD programmes’ operations, infrastructure, partnerships, and funding alongside population displacement and limited access to conflict regions impede the programmes’ ability to apply interventions. Examples of this include (1) Uganda’s eventually successful GWD elimination programme which suffered delayed implementation of interventions, loss of personnel, and importation of cases due to civil conflicts [59], and (2) Mali’s trachoma efforts which prevailed despite a military coup d’état leading to socio-political instability and withdrawal of donor support [107]. More recently, conflicts and instability are adversely impacting NTD research in Sudan [237] and overall health infrastructure in the Democratic Republic of the Congo [238]. Health diplomacy, as practiced by the Carter Center, is important for achieving and sustaining health victories and progress towards NTD elimination (S4a Table). This work should aim for peaceful power transitions where health provision is prioritised. As conflict-caused displacement of people may cause NTDs to spread to new geographical areas [206], healthy regional diplomatic relations can ensure robust prevention, preparation, and responses to these challenges. Indeed, adjacent countries and regions cannot eliminate NTDs in isolation of each other, and can benefit from shared regional One Health approaches [215]. Cross-border collaboration and initiatives, such as the Pan African Tsetse and Trypanosomiasis Eradication Campaign against HAT [239], may reduce spread of cases across borders and ensure no infection reservoirs remain unaddressed, thus helping sustain elimination progress. This may be particularly important in Africa, where 49 countries, and possibly more, are each affected by multiple NTDs (Fig 2).

Overall, we recommend that in ongoing and future NTD control and elimination initiatives, countries systematically carry out dedicated efforts and tailored interventions through to post-elimination stages to prevent resurgence. More must be done to facilitate increased integration of programmes and transition away from siloed approaches. To allow for integration into PHC, health systems must first be strengthened. The eight elimination strategies presented in this review should be integrated and One Health approaches prioritised for cross-cutting impact. Country ownership, leadership, and accountability are essential throughout the entire elimination process and beyond. International partners must continue to support integration and health system strengthening, while both domestic and international funding should be increased. However, reducing reliance on external support is crucial. Lessons from past efforts should be carefully considered to avoid the repetition of mistakes; this includes maintaining prioritisation, ensuring adequate resources, practicing health diplomacy, fostering regional collaboration, and avoiding complacency.

A key strength of this review is its originality: the synthesised evidence from successful NTD elimination programmes presented here fills a gap in literature as similar work has not been previously published. The literature review was conducted in a rigorous, reproducible and comprehensive manner facilitated by the inclusion of both published and grey literature. A conservative approach to data extraction was employed: only explicit statements of information were included, and no data or information was inferred. An exception to this was where elimination programme integration level was assumed to be stand-alone when no explicit information on integration was provided. The timeliness of this study is highlighted by the pressing challenge of achieving the WHO’s global NTD targets by 2030, including 100 countries eliminating an NTD, and yaws and Guinea worm disease being eradicated. Currently, 44 more countries must eliminate one or more NTDs to reach the target of 100 countries by 2030. The blueprint for NTD elimination presented in this review provides actionable insights and lessons for governments, policymakers, and international stakeholders to consider in expediting the control and elimination of NTDs.

Despite the comprehensiveness of this review, there were some missing details on the programmes in the countries analysed, including incomplete data on types of support provided by partners and historical programmes. Furthermore, data on programme integration was often fragmented, hence forming a complete picture of this was challenging. In the data syntheses in this review, these data gaps reflect absence of evidence rather than evidence of absence. A further methodological limitation is that Ministries of Health, partner organisations, or other stakeholders were not directly contacted for information. The search strategies employed to retrieve published literature contained terminology only in English, hence, only publications originally in or translated into English were retrieved, possibly limiting comprehensiveness. Furthermore, literature searches and screening were conducted by two individuals within a short time frame, and second literature searches and screening were only conducted on PubMed. Factors limiting the reliability of included grey literature sources include variability in reporting standards, lack of peer-review, and potential biases; these were mitigated by choice of credible organisations as sources and cross-checking findings where possible. Finally, as the latest eliminations of gHAT in Chad and Guinea, LF in Brazil and Timor-Leste, trachoma in Pakistan, Vietnam, India, Mauritania and Papua New Guinea, onchocerciasis in Niger, and leprosy for the first time in Jordan, were all validated or verified after March 2024 [13–18,240–244], their elimination processes are excluded from this review.

To overcome the data gaps which have limited this review, future research and elimination programme documentation efforts should focus on accurately and consistently recording programmatic functioning of elimination programmes. Standardised methodology for countries to document and report NTD control and elimination efforts is required. The move by the WHO encouraging the collection of gender-disaggregated data and more granular data through precision infection mapping will improve the quality of data collected, and consequently produce better targeted elimination efforts where infection hotspots occur. Furthermore, cost-effectiveness evaluations should be conducted to define the most economical approaches to NTD elimination, especially in resource-limited countries. As more NTDs approach elimination, the cost-effectiveness of interventions previously used in high-prevalence settings may diminish, making these evaluations increasingly important [245]. The WHO’s road map operational plan 2025–2030 is planned to include estimated costs of certain NTD-targeting interventions to facilitate resource mobilisation and optimisation [12]. Future study should also determine total costs of elimination programmes to facilitate efficient budgeting. Additionally, while this study provides a foundational blueprint, it did not delve into the specific factors that differentiate countries that have successfully eliminated more than two NTDs, such as Togo, Benin and Ghana, from those facing delays in elimination. Future work should aim to systematically examine these contrasting experiences to identify critical programmatic, contextual, and operational factors that contribute to success or hinder progress. Such in-depth comparative analyses could offer valuable insights for tailoring interventions, optimising resource allocation, and informing policy decisions to accelerate elimination efforts in struggling settings.

### Conclusions

Taken the lessons learnt from both the successful and failed NTD elimination efforts to date, to accelerate NTD elimination countries must have ownership over elimination efforts and sustain their commitment to efforts long-term. Dedicated efforts and the use of tailored and cross-cutting strategies, including the eight presented in this review, are essential to success. Increased integration of programmes to cover multiple NTDs and other diseases, and programme mainstreaming into national health systems, are required for increased programme interdisciplinarity, resilience and efficiency. Crucially, advancing elimination efforts demands a shift towards cross-cutting One Health approaches that bridge human, animal and ecosystem health; these include water, sanitation and hygiene (WASH), integrated vector control, health system strengthening, and ecosystem-based interventions. Addressing underlying social and environmental determinants of health via systems thinking must be prioritised. Additionally, there must be increased domestic and international investment towards NTD elimination initiatives, with partner organisations committing to supporting integrated One Health initiatives with increasingly domestically sourced funding. Exercising health diplomacy to create regional collaborations is necessary to eliminate infection reservoirs and reduce geographical spread of NTDs, particularly in the context of increasing global mobility, conflicts, and climate-related health challenges. Lessons from failed historical elimination efforts must be learnt and remembered to avoid repetition of mistakes.

Moreover, gaps in data and unavailability of comprehensive documentation continue to hinder a full understanding of global elimination efforts. Moving forward, systematic and standardised reporting and improved collaboration between national governments and international partners will be critical to sustaining progress. Addressing these gaps will enhance future elimination initiatives, ensuring more efficient use of resources and supporting the achievement of the WHO’s 2030 NTD control, elimination, and eradication goals. For NTDs such as schistosomiasis which already exhibit features of successful elimination programmes identified here, it is also critical to understand why they have not yet been eliminated in a single country. Identifying and addressing these elimination bottlenecks will ensure no NTD is left behind in global NTD elimination efforts.

## Supporting information

S1 Table Abbreviations, acronyms and their definitions used in figures and tables of this review. (S1_Table.DOCX)

S2 Table Additional detailed information on the employment of the eight NTD elimination strategies across the 50 countries [246–252]. (S2_Table.DOCX)

S3 Table Partners having supported one or two elimination programmes. (S3_Table.DOCX)

S4 Table **(a)** Detailed aspects and examples of NTD elimination support provided by the WHO and Carter Center, presented in Fig 7. **(b)** Detailed aspects and examples of NTD elimination support provided by some of the partners presented in Fig 7 [253–257].
(S4_Table.DOCX)

S5 Table Additional details of failed historical NTD control efforts [258]. (S5_Table.DOCX)
