# Supplementary material for: Unlocking the blueprint to eliminating neglected tropical diseases: A review of efforts in 50 countries that have eliminated at least 1 NTD
Source: PLoS Negl Trop Dis. 2025 Sep 4;19(9):e0013424. doi: 10.1371/journal.pntd.0013424 (PMC12410759; doi:10.1371/journal.pntd.0013424)
Supplement: S1 Table — (S1_Table.DOCX) [file pntd.0013424.s001.docx]

**Table S1: Abbreviations, acronyms and their definitions used in figures and tables of this review.**

| Abbreviation / Acronym | Definition |
| --- | --- |
| ABATE | commercial name of larvicide used in efforts against GWD |
| COMBI | communication for behaviour impact |
| DDT | dichlorodiphenyltrichloroethane (insecticide) |
| DEC | diethylcarbamazine (drug) |
| IEC | information, education and communication |
| IRS | indoor residual spraying |
| KAP | knowledge, attitudes and practices |
| LLIN | long-lasting insecticide-treated nets |
| MDA | mass drug administration |
| PCR | polymerase chain reaction |
| PEP | post-exposure prophylaxis |
| PHC | primary health care |
| SAFE | Surgery, Antibiotics, Facial cleanliness and Environmental improvements |
| TAS | transmission assessment survey |
