# Supplementary material for: Unlocking the blueprint to eliminating neglected tropical diseases: A review of efforts in 50 countries that have eliminated at least 1 NTD
Source: PLoS Negl Trop Dis. 2025 Sep 4;19(9):e0013424. doi: 10.1371/journal.pntd.0013424 (PMC12410759; doi:10.1371/journal.pntd.0013424)
Supplement: S2 Table — (S2_Table.DOCX) [file pntd.0013424.s002.docx]

**Table S2: Additional detailed information on the employment of the eight NTD elimination strategies across the 50 countries.**

| Case detection and disease monitoring |
| --- |
| As part of this strategy, prevalence mapping has been carried out to establish the distribution and level of NTD prevalence within countries (48–60,62,64,66–68,73,76–91,94–97,99–113,116–118,125–179). This mapping has been achieved via questionnaires, historical record examinations, rapid assessments and prevalence surveys, and has usually been conducted prior to other interventions. This has allowed for determining the required intensity and localisation of subsequent interventions. Surveillance, which involves ongoing collection and analysis of NTD data to monitor case numbers and transmission (246), has been performed via detection, reporting and investigations of cases, and entomological surveying where data is collected on insect vectors (48–60,62,64,66–68,73,76–91,94–97,99–113,116–118,125–179). Surveillance can be 1) active, where proactive case searches are performed village-by-village or door-to-door by e.g. mobile teams, or 2) passive, where symptomatic patients present themselves to fixed health facilities (133,138,187). Campaign-natured screenings have also been conducted within at-risk communities (102,106,172). |
| Case management |
| Case management includes trachoma blindness prevention surgeries performed as part of SAFE strategy: these surgeries were performed free-of-charge at least in some cases (72,103,108,109,111,112,166,168,170,172,247,248). Case containment, defined as rapid case detection and care to prevent onwards transmission, was done particularly with GWD (50–52,58,59,70,133,133,249). To do this, some countries used specific case containment centres where infected persons are provided with free lodging and treatment (50–52,58,59,127,130,132,133). Morbidity management and disability prevention, particularly in LF elimination efforts, has involved health worker home visits, patient treatment at health centres, hydrocoele surgeries, lymphoedema health education, provision of self-care guides and morbidity management kits, all of these usually free-of-charge (50,76–80,84,88,90,91,148,149,155). |
| Health system capacity building and strengthening |
| Training of medical professionals, health staff, community workers, volunteers, and field workers has been done as part of elimination efforts in at least 34 countries across NTDs (48,51,53,55–57,59,76–79,83,84,86,87,90,91,101,103,105,108,112,117,126,128,130,134,136,137,146,148,149,154,163,167,168,172,178). Evaluation of elimination programme performance was performed for GWD elimination efforts in e.g. Pakistan (58). Nigeria and Uganda have also monitored the work of volunteers and health workers, and replaced those with unsatisfactory performance (59,133). In Benin and Ghana, women were actively recruited to be involved in GWD elimination efforts as they often had first-hand knowledge of current developments (51,128,250). Knowledge, Attitudes and Practices (KAP) surveys have been conducted to detect gaps in NTD-related knowledge within affected communities (87). Information obtained via KAP surveys has allowed for design and improvement of social mobilisation campaigns (87,95). Clinical and operational research has been conducted as part of Bangladesh’s VL elimination programme (117): this has entailed drug and vector control trials, as well as establishment of a VL research centre. Integration, on the other hand, was a key part of trachoma elimination organisation in Saudi Arabia and Oman: Saudi Arabia integrated an eye care programme into primary health care provision (125), while Oman integrated surveillance into both the national school programme and existing health care services (105). Advocacy for the elimination of yaws has been conducted in India (118). |
| Community engagement and education |
| Social mobilisation, aiming to educate and engage NTD-affected communities, encompasses a variety of activities, including awareness campaigns to improve participation in and acceptance of elimination interventions, and education on safety and efficiency of interventions such as MDA or vector control (51–60,76,77,79–81,84,86,88,91,94,95,99,103,105,108–112,117,118,126,128,130,133,136,140,141,147–149,151–153,155,160,164,167,168,170,172). Additionally, particularly in the context of GWD elimination, cash rewards have been offered to community members for detecting, reporting, and containing cases (49,52,56,58,59,70,137). |
| Preventive chemotherapy |
| As part of SAFE strategy’s component A, antibiotic MDA has been used to eliminate trachoma (72,103,109,111,112,172,174,184,251). Several rounds of MDA to combat LF have been carried out by community health workers, volunteers, or teachers (50,77–82,84,86,87,89,91,148–150,154,157). Drugs have been provided to eligible people by door-to-door distribution, or in schools, churches, community halls or other community spaces. For onchocerciasis, high-coverage MDA has been carried out by health workers once, twice or four times a year, for several years overall (94,95,97,99). For some countries eliminating LF or onchocerciasis, drug taking has been directly observed (81,86,87,95,99). Various types of MDA-related surveys have also been performed for both LF and onchocerciasis to evaluate the impact of MDA and determine whether MDA could be ceased. To combat yaws, India did selective mass treatment of infected individuals and their contacts with penicillin (179). |
| WASH (water, sanitation and hygiene) |
| In GWD elimination efforts, nylon or cloth filters have been used for drinking water to prevent ingestion of Guinea worm larvae-carrying copepods, as this would lead to infection (50–53,55,56,58–60,62,68,126,128,131,133,136). Additionally, safe water access and supply for communities has been improved by building and rehabilitation of wells as well as by advocacy with water organisations (64,134) . The implementation of the SAFE strategy’s F and E components (Facial cleanliness and Environmental improvements) has involved facial hygiene promotion and education, upgrades to living conditions, enhancements to sewage disposal by latrine building, and improvements to water supply via borehole and water pump provision and construction of wells (101,103–105,108–112,165–168,170,172–174). Iran, which did not have a dedicated elimination programme for trachoma, relied partly on general implementation of WASH for elimination (105). |
| Vector control |
| In 11 countries which have eliminated GWD and implemented vector control, temephos-containing ABATE larvicide has been applied at regular intervals, e.g. monthly (51–53,57,59,60,62,67,68,126,128,134–136). In vector control against gHAT, insecticide-impregnated Tiny Targets cloth panels, which attract and eliminate tsetse flies, have been used in Uganda and Côte d’Ivoire to reduce tsetse fly population densities (138,142). Vector control has also been implemented against LF in several countries (84,91,149,154). In Malawi, Yemen, Laos, and Vanuatu, malaria vector control interventions, including use of insecticide-treated nets and indoor residual spraying, where insecticide is applied to indoor walls and ceilings (252), have contributed to LF elimination (80,87,91,151). Similarly, Bangladesh’s VL elimination programme involved integrated vector management, a method combining several vector control interventions such as use of insecticide-treated bed nets and indoor residual spraying (117,177). |
| Veterinary public health |
| In Mexico, annual mass dog vaccination campaigns were carried out to achieve high immunisation levels (100). Additionally, cats and dogs were sterilised (162). In Rwanda, animals infected with *Trypanosoma brucei rhodesiense* have been treated since cattle and wildlife form a major reservoir (146,147). |
