# Supplementary material for: Unlocking the blueprint to eliminating neglected tropical diseases: A review of efforts in 50 countries that have eliminated at least 1 NTD
Source: PLoS Negl Trop Dis. 2025 Sep 4;19(9):e0013424. doi: 10.1371/journal.pntd.0013424 (PMC12410759; doi:10.1371/journal.pntd.0013424)
Supplement: S3 Table — (S3_Table.DOCX) [file pntd.0013424.s003.docx]

**Table S3: Partners having supported one or two elimination programmes.**

| Names of 53 partners having supported one elimination programme |
| --- |
| AIMES Afrique, Arab Fund for Economic and Social Development, Asian Development Bank, AusAID, Australian NGO Cooperation Program, AVSI Foundation, CARE Cameroon, Carlos Slim Foundation, Children’s Investment Fund Foundation, Coalition for Operational Research on Neglected Tropical Diseases, Collegio Universitario Aspiranti e Medici Missionari (now Medici con l’Africa), Conrad N. Hilton Foundation, Drugs for Neglected Diseases Initiative, Eisai Co. Ltd, ELMA, Commonwealth Fund, FIND, German Leprosy Relief Association, Gilead Sciences, governments of Canada/Denmark/ Korea/Kuwait/Norway/Oman/Saudi Arabia/Sweden/United Arab Emirates/United States, Hassan II Ophthalmology Foundation, Himalayan Cataract Project, IMAworld, Institute for One World Health, International Centre for Diarrhoeal Disease Research, Islamic Development Bank, Islamic Relief, James Cook University, Japanese businessmen’s consortium Keidanren, KalaCORE, Korea Disease Control and Prevention Agency, Margaret A. Cargill Foundation, Médecins Sans Frontières Holland, Medical Research Council UK, Memorandum of Understanding 2005 (Bangladesh, India and Nepal), Nepal Netra Jyoti Sangh, PATH, Sasakawa (Global 2000), Sight For All, Soap Aid, Virgin Unite, Yakubu Gowon Centre, Yemen Leprosy Elimination Society, Yeux du Monde |
| Names of 10 partners having supported two elimination programmes |
| Fred Hollows Foundation, London School of Hygiene and Tropical Medicine, Pacific Community, Pan African Tsetse and Trypanosomiasis Eradication Campaign, Queen Elizabeth Diamond Jubilee Trust, Task Force for Global Health, Tropical Data, Trypa-NO! Partnership, Water Aid, World Bank |

References: (48–50,52,55,59,64,65,77,79–81,84,88,91,101,103,110–112,116,117,140,152,168,169,172–174,177,184,188,189,195,198)
