# Supplementary material for: Unlocking the blueprint to eliminating neglected tropical diseases: A review of efforts in 50 countries that have eliminated at least 1 NTD
Source: PLoS Negl Trop Dis. 2025 Sep 4;19(9):e0013424. doi: 10.1371/journal.pntd.0013424 (PMC12410759; doi:10.1371/journal.pntd.0013424)
Supplement: S4 Table — (a) Detailed aspects and examples of NTD elimination support provided by the WHO and Carter Center, presented in Fig 7. (b) Detailed aspects and examples of NTD elimination support provided by some of the partners presented in Fig 7 [253–257]. (S4_Table.DOCX) [file pntd.0013424.s004.docx]

**Table S4a: Detailed aspects and examples of NTD elimination support provided by the WHO and Carter Center, presented in Fig 7.**

| The World Health Organization (WHO) |
| --- |
| The WHO is an important global collaborator and driver of progress in NTD elimination efforts: it creates elimination initiatives and milestones, establishes standard guidelines, plans and endorses strategies, and monitors elimination progress (4). The WHO sets the criteria for NTD elimination statuses, and provides countries with official elimination validation or verification (31). The WHO’s second and most recent NTD road map guides NTD control, elimination, and eradication efforts globally, and sets goals for 2030 (4). An example of the WHO’s support for NTD elimination is its collaboration with the pharmaceutical company Gilead Sciences to donate liposomal amphotericin B, a first-line VL drug, to Bangladesh’s VL elimination programme (116). The WHO has also managed GSK’s albendazole donation to at least six LF preventive chemotherapy programmes (77,79,84,86,87,148,149). Additionally, the Global Programme for Eliminating LF was launched by the WHO in 2000; this served as the global initiative and model programme for several national LF elimination programmes (76,80,154). WHO-sourced funding has also been important, as in the case of Togo’s HAT elimination efforts which were solely funded by the WHO (50). The WHO’s regional office for the Americas, PAHO, has supported onchocerciasis and rabies elimination efforts in Latin America (94,161,253). |
| The Carter Center (CC) |
| The CC’s work has included global leadership, health diplomacy and operational support. CC has been most heavily involved in GWD elimination, having helped 14 countries to achieve this (50,51,53,56,59–65,129,135,136). CC has also been leading the global Guinea Worm Eradication Programme since 1986 (66,67,254). Examples of CC’s support include facilitation of GWD radio announcements in Benin (51), organisation of National Guinea Worm Days in Cameroon (62), as well as distribution of water filters and management of ABATE larvicide application in Central African Republic (68). CC has also recruited women to assist in GWD interventions in Ghana (128), and supported the establishment of 17 GWD containment centres in Togo (255). In Nigeria, CC recruited a former head of state to advocate for GWD efforts (134), and in Pakistan coordinated with the president and prime minister to launch Pakistan’s national GWD elimination programme (66). CC has helped mediate a conflict between Uganda and Sudan which had delayed Ugandan GWD elimination (59). Additionally, CC has lead the Onchocerciasis Elimination Program for the Americas (OEPA) since 1996 (256), and supported Colombia, Ecuador, Guatemala, Mexico in onchocerciasis elimination (94,96,158,191). CC has supported Ghana and Mali in trachoma elimination (104,184). |

**Table S4b: Detailed aspects and examples of NTD elimination support provided by some of the partners presented in Fig 7.**

| GSK, Pfizer, Merck and BASF |
| --- |
| Pharmaceutical companies GSK, Pfizer and Merck, and the chemicals company BASF, have supported elimination programmes and efforts via product donations. GSK donated albendazole to preventive LF chemotherapy in 10 countries (50,77–81,84,86,87,148). Azithromycin antibiotic was donated by Pfizer to trachoma elimination efforts in eight countries (101,103,104,109,112,169,170,173). Merck, on the other hand, has been donating ivermectin (Mectizan) via the Mectizan donation programme to four countries in onchocerciasis elimination efforts, and Malawi in LF elimination efforts (80,94,158,189,191). BASF has donated ABATE larvicide to Guinea worm disease elimination efforts in at least 10 countries (51–53,57,59,60,62,68,126,128,134,136). |
| PacELF |
| PacELF, established in 1999, has united 22 Pacific island nations in the endeavour of eliminating LF (78,193). Eight countries of the Pacific region have achieved LF elimination with PacELF’s support (78,81,86,87,157,193,194) (Fig 7). PacELF has developed frameworks and policies for LF elimination, and it promotes preventive chemotherapy and case management as the main strategies for LF elimination (157,193,203). PacELF itself has been supported by e.g. WHO, JICA, CDC and GSK (193). |
| BMGF, CDC, HDI, HKI, ITI, JICA, LSTM, PC, RC, SS, USAID |
| JICA has assisted in the establishment of a VL research centre in Bangladesh (117), and has also facilitated the procurement of diethylcarbamazine for LF preventive chemotherapy programmes on Cook Islands and Tonga (78,86). ITI, on the other hand, has managed Pfizer’s azithromycin donation to trachoma elimination efforts in the Gambia, Malawi, Nepal, Morocco and Vanuatu (103,109,112,168,173). LSTM has been involved in the development of the Tiny Targets vector control method, important for facilitating gHAT elimination in Uganda (142,257). HDI, BMGF, USAID and CDC have supported Togo’s LF elimination programme, which had no operational budget and sourced funding entirely from partners (50). HKI and Sightsavers have supported Mali’s trachoma and LF elimination efforts (184). In-the-field operational support was provided to GWD elimination and control efforts by Peace Corps volunteers in Cameroon, Nigeria and Togo (52,133,255). In Ghana, this was done by Red Cross volunteers (196). |

Table 4 contains the full names of the here abbreviated partners.
