# Supplementary material for: Unlocking the blueprint to eliminating neglected tropical diseases: A review of efforts in 50 countries that have eliminated at least 1 NTD
Source: PLoS Negl Trop Dis. 2025 Sep 4;19(9):e0013424. doi: 10.1371/journal.pntd.0013424 (PMC12410759; doi:10.1371/journal.pntd.0013424)
Supplement: S5 Table — (S5_Table.DOCX) [file pntd.0013424.s005.docx]

**Table S5: Additional details of failed historical NTD control efforts.**

| Lack of effective interventions and/or their insufficient intensity |
| --- |
| Upon cessation of Bangladesh’s historical malaria vector control, which had collaterally helped control VL, VL resurged as no suitable replacement intervention was adopted (176,177,204). Similarly, an effective intervention in the form of a safe and effective drug lacked in early onchocerciasis control efforts (96). To alleviate clinical case severity, historical onchocerciasis control programmes in Ecuador, Guatemala and Mexico performed nodulectomies where nodules containing adult worms are surgically removed from infected individuals (95–97,99,159). At least in Ecuador, these were not effective enough to control onchocerciasis (99), and interruptions of transmission were only achieved once rounds of MDA began. In historical LF control programmes in Egypt and Maldives, selective case treatment as opposed to MDA was performed (79,154). This likely contributed to programme failures as selective treatment lacks the intensity of MDA treating and preventing cases in entire groups or populations (121). |
| Lack of prioritisation / deprioritisation |
| The Mexican government began aiming for rabies control in the 1970s, yet this was not achieved then as other disease programmes were prioritised (160). Equatorial Guinea’s historical HAT control programme 1930-1968 failed for a similar reason (73). Despite this programme’s success in lowering case numbers, cases resurged as, upon the country gaining its independence, acute independence-related issues took priority. Similar resurgences occurred in several African countries as their health services were disrupted by socio-political changes or instability around mid-1960s (258). |
| Deprioritisation, complacency, and poor integration into PHC |
| In Uganda, historical efforts against HAT came close to achieving elimination by mid-1960s. Unfortunately, complacency and subsequent deprioritisation of efforts followed, along with eventual resurgence (142).  In India’s historical yaws elimination efforts (118,178), complacency set in once yaws prevalence had decreased notably thanks to a campaign launched in 1952. This led to discontinuation of efforts and disease re-emergence in the late 1970s. India’s historical yaws programme also suffered from poor integration of interventions into PHC (178). After cases decreased post-1952, PHC was to be responsible for continuing passive surveillance and residual case treatment (118,178). However, areas in which PHC was to take this responsibility had no PHC provision to begin with (178). This also contributed to resurgence. |

For acronyms, see Supplementary Table 1.
